# Physical environmental opportunities for active play and physical activity level in preschoolers: a multicriteria analysis

**DOI:** 10.1186/s12889-022-12750-8

**Published:** 2022-02-17

**Authors:** Juliana Nogueira Pontes Nobre, Rosane Luzia De Souza Morais, Bernat Viñola Prat, Amanda Cristina Fernandes, Ângela Alves Viegas, Pedro Henrique Scheidt Figueiredo, Henrique Silveira Costa, Ana Cristina Resende Camargos, Marcus Alessandro de Alcantara, Vanessa Amaral Mendonça, Ana Cristina Rodrigues Lacerda

**Affiliations:** 1 Centro Integrado de Pós-Graduação e Pesquisa em Saúde (CIPq-Saúde), Universidade Federal dos Vales do Jequitinhonha e Mucuri (UFVJM), Diamantina, Minas Gerais Brazil; 2 Faculdade de Fisioterapia, Universidade Federal dos Vales do Jequitinhonha e Mucuri (UFVJM), Diamantina, Minas Gerais Brazil; 3 Instituto de Ciência e Tecnologia (ICT - UFVJM) e SaSA, Universidade Federal dos Vales do Jequitinhonha e Mucuri (UFVJM), Diamantina, Minas Gerais Brazil; 4 Faculdade de Fisioterapia, Universidade Federal de Minas Gerais (UFMG), Belo Horizonte, Brazil

**Keywords:** Outdoor activities, Active play, Environmental, Outdoor recreation

## Abstract

**Background:**

Active play opportunities seems to influence the level of physical activity during childhood. However, a gap remains about which environmental opportunities including the daycare physical environment could have a positive impact on the level of physical activity in preschoolers.

**Objectives:**

(1) To develop an index to measure the environmental opportunities of free active play for preschoolers of middle-income countries; (2) to check the relationship and contribution of the index to explain objectively the level of physical activity.

**Methods:**

A quantitative, cross-sectional, exploratory study with 51 preschool children. The established criteria for the index according to the literature were: (1) Outdoor time on typical days of the week. (2) Outdoor time on a typical weekend day. (3) The presence of internal space and external environment in the child’s home that allows playing. (4) Presence of patio with space for games at the school. (5) Presence of a playground with a toy at the school. We applied multi-attribute utility theory for the determination of the multicriteria index of physical environmental opportunities. Pearson’s correlation analysis and simple linear regression were used to verify the association between the index and the physical activity level.

**Results:**

The index showed a positive correlation with the level of physical activity, e.g., the average time of MVPA (r = 0.408, *p* = 0.003). The univariate linear regression demonstrated that the quality of physical environmental opportunities for physical activity explained 20% of the preschooler’s classification as active and 16% of the time in moderate to vigorous physical activity (*p* < 0.001).

**Conclusion:**

Physical environmental opportunities for active play have a positive effect on physical activity in preschoolers and should be encouraged in different social segments.

## Background

Physical activity (PA) for children is the basis for healthy growth. Lifestyle habits, such as PA participation, developed throughout childhood affect adolescence and adulthood [1]. Sufficient PA in early childhood (under 5 years old), especially at moderate to vigorous physical activity (MVPA) [2], can promote immediate metabolic benefits in blood pressure, lipid profile [3], reduce the risk of disease and weight gain [1] and improve social, emotional, cognitive aspects [4]. Finally, evidence points that PA in preschoolers can promote the development of important motor skills for success in motor tasks and subsequent engagement in sports influencing a healthy lifestyle in adulthood [1, 5, 6]. Despite this, a recent study showed an increase in sedentary behavior and a reduction in PA in preschoolers [7]. In addition, a growing number of children worldwide are failing to perform the minimum of recommended PA to acquire a healthy lifestyle [7, 8]. As PA levels often decrease throughout the school phase of children and adolescents [9], preschool phase is considered a crucial period to establish healthy PA habits throughout childhood [10].

Although previous study suggested that children’s PA levels are mainly influenced by genetic factors [11], evidences also point out the influence of family [12, 13], economic classification [13] and personal factors on PA levels [14–18]. In this setting, sex and age were determinants of personal factors such that male [19] and, therefore, male preschoolers, older [19] and exposed to the outdoors [20] had higher PA intensities. In addition, socioeconomic disparities related to the economic classification of households (including comfort items in working order in the household, housing security conditions, neighborhood, the stretch of street of the household, and the householder’s education) [21] may also affect the PA levels [22].

Previous studies highlighted the home environment as an important space for the promotion of PA in preschoolers [14, 16, 17]. Evidence pointed out important facilitators for PA, among which are the home environment, the preschool environment, and their interactions reciprocal between the child and the physical environment [23].

The home is a behavioral environment in which children spend a great deal of time and understanding the PA facilitators in this physical environment is necessary [14], Briefly, studies showed that playing outdoors at home can be an important source of PA for many preschoolers [24–26]. Elements of the home outdoor environment, e.g., presence and attributes of the yard, have been associated with increased PA in preschoolers [27, 28]. However, not only the presence of a yard, but also how often they frequent the yard seems to influence the level of PA and reduce the sedentary time [26]. Moreover, studies reinforce the association between outdoor time on weekdays and weekends with PA especially in preschoolers [24, 29, 30]. On the other hand, the internal home environment can inhibit or stimulate PA in preschoolers, limited internal space, e.g., apartments lacking spaces, inhibit active opportunities [23, 31, 32], whereas larger spaces seem to benefit children’s PA [32, 33]. Studies demonstrated that preschoolers classified as highly active compared with insufficiently active are often active in indoor environments [34] reinforcing the idea that indoor environment offers untapped potential to promote and support PA [35].

The daycare physical environment has also the potential to influence PA and general health and the development of children under care [3, 35, 36]. Thus, we should keep in mind the daycare physical environment should be an ideal setting for promoting PA due to the unique opportunity for structured PA for all children, regardless of children’s characteristics and parents’ behaviors, attitudes, and resources [19]. In this sense, a greater space per child and open play areas could increase PA in children attending daycare centers [37]. The presence of portable play equipment and a playground [20] were also associated with greater PA in preschoolers [37, 38]. Further investigation into the relative value of outdoor game designs, as well as the presence and quality of individual characteristics of the physical environment, e.g., free space, leisure equipment, vegetation, paths and shade, should be clarified to identify the physical environmental characteristics that best promote PA in the daycare physical environment [39, 40].

The historical process of the daycare physical environment took place differently worldwide. Thus, in certain middle-income countries is common to find physical spaces restricted and inappropriate for children [41]. Regulatory and operational policy frameworks for the school environment have received little attention [22] despite evidence about the importance of physical space and parks in promoting children’s PA [19, 20]. Added to this reality, there are gaps in the understanding of what are the facilitating opportunities for the PA level of preschoolers that address both the home environment and the daycare center [22]. Despite evidence of the importance of the environmental factor for PA in the daycare environment [20], and in the home environment [24–26, 28], there is currently a lack of discussion about existing measures [22] since studies consider different methodologies [20, 22, 23]. Finally, understanding that child development is a multifactorial construct resulted from the child’s reciprocal interactions with the physical environment [42], multicomponent models have been encouraged [43] to understand how strategies that increase the PA intensity of preschoolers can combine multiple factors like the home and daycare environment [42, 43]. Thus, the aims of our study were (1) To develop an index to measure the physical environmental opportunities of free active play for preschoolers of middle-income countries. (2) To check the relationship and contribution of the index to explain objectively the level of PA.

## Materials and methods

### Study design

This is a quantitative, exploratory, cross-sectional study, approved by the Research Ethics Committee of the Universidade Federal dos Vales do Jequitinhonha e Mucuri-UFVJM (Protocol number: 2,773,418), with the written informed consent of those responsible and the consent of the participants. Data collection took place from July to December 2019.

### Participants

From 11 public schools in a Brazilian municipality, the participants who accepted were from 9 schools. Sample size was estimated using the OpenEpi software, version 3.01, following a study with similar design [43]. For this, a prevalence of 3% of Brazilian preschoolers who meet the PA guidelines recommended by the WHO [44]was considered, with a desired accuracy of 10%, a confidence interval of 95% and an effect size of 1 [45]. Considering a population of 1241 children enrolled in the Brazilian municipality studied [46], and an adjustment for possible sample losses of 10%, the sample size resulted in 51 preschool children.

Exclusion criteria were premature and low birth weight babies; babies with complications in pregnancy and childbirth; babies with signs of malnutrition or diseases that interfered with growth and development. Children with any condition that interfered with cognitive and motor development were also excluded.

### Instruments

For the characterization of the participants, a questionnaire was developed to collect data on the child’s birth and health. In addition, the shift the child studies (full, partial), the mother’s education and the child’s family’s economic level were checked-using questionnaires suitable for preschoolers. The Brazil economic classification criterion from the Brazilian Association of Research Companies (ABEP) was used to verify the economic level of families. This is a questionnaire that stratifies the general economic classification resulting from this criterion from A1 (higher economic class) to D-E (lower economic class) [47].

The PA level was measured using an accelerometer (Actigraph®- Model GT9X); for a period of 3 days [48], for a minimum of 570 min a day [49], which is considered suitable for preschoolers [48]. Accelerometers were initialized and analyzed using 5-s epochs. In all analyses, consecutive periods of ≥20 min of zero counts were defined as non-wear time [50], with a sampling rate of 60 Hz. The accelerometer was positioned on the right side of the hip to capture accelerations and decelerations of the body and determine objective measurements of gross acceleration and intensity of physical activity [50]. A trained researcher placed the device on the child’s right hip at 7 am and the parents were instructed to withdraw at 19 pm. Pediatric cutoff points validated for preschoolers, with score values, classify as sedentary intensity (0 to 819 counts/m), light intensity (820 to 3907 counts/m), moderate intensity (3908 to 6111 counts/m) and vigorous intensity (above 6612 counts/m) [51]. For this study, the child’s mean time at these intensities was used. The classification adopted for “active” or “insufficiently active” was established according to the WHO, which considers an active child to be one who has a PA of at least 180 min/day, with a minimum of 60 min/day in MVPA [52]. The accelerometer data was initially downloaded using ActiLife Software (version 5.10) and then analyzed using custom Excel macros.

The quality of the environment in which the child lives was assessed using the Early Childhood Home Observation for Measurement of the Environment (EC_HOME) [53] . The EC_HOME is applied through observation and semi-structured interviews during home visits, standardized for children aged 3 to 5 years. The instrument contains 55 items divided into 8 scales: I-Learning materials, II-Language stimulation, III-Physical environment, IV-Responsiveness, V-Academic stimulation, VI-Modeling, VII-Variety, and VII-Acceptance. Each item in each domain was scored in a dichotomous manner (0 or 1); with the maximum score of the instrument 55 points (higher scores reflected better evaluation in each domain). Of note, the sum of the gross scores of the subscales was classified in the following ranges: Upper Fourth (values between 46 and 55 points), Middle half (values between 30 and 45 points), and Lowest Fourth (values between 0 and 29 points). For analysis, the sum of the raw scores of the subscales was used. For the elaboration of the multicriteria index of physical environmental opportunities, we used two items of the subscale III of the referred instrument, which assesses, among others, the presence of a yard and the internal physical environment of the house considering 30m^2^ per inhabitant. The HOME Inventory has been used worldwide to evaluate the home environment in both international [54] and transcultural studies [55], presenting psychometric characteristics investigated in Brazilian preschoolers sample- analysis of internal consistency satisfactory for the total scale (Cronbach’s Alpha = .84 for the 55 items) [56].

The outdoor time questionnaire proposed by Burdette et al. [57], evaluated the daily time of participation in games and outdoor games and sedentary behavior (daily time watching television) at home. The parents completed the questionnaire in relation to the child’s behavior on a typical day of the week and on a typical day of the weekend, considering three different periods of the day. Each period the time reported by the parents was recorded and the sum of this time outdoors in minutes calculated. This questionnaire was validated for Brazilian preschoolers [58].

The quality of the school environment was assessed using the Early Childhood Environment Rating Scales (ECERS) [59], which contain inclusive and culturally sensitive indicators for many items. The scale consists of 43 items organized into 7 subscales (1-Space and Furnishings, 2-Personal Care Routines, 3-Language and Literacy, 4-Learning activities, 5-Interactions, 6-Program Structure, 7- Parents and staff). Each quality indicator was marked, considering its presence or absence in each collective environment (classroom), with the items scored from 1 to 7. The final score of the scale is given by the mean of the seven subscales. It is an ordinal, increasing scale, from 1 to 7, the interpretation of quality being 1: inadequate; 3: minimal (basic); 5: good; 7: excellent. For the elaboration of the study index, two items from subscale 1 were used, which included the presence physics and use of a park and toys in addition to the school space. This questionnaire is a well-known international instrument translated to Portuguese [60] and used in different Brazilian studies including preschoolers [60, 61]. Of note, the instrument presents psychometric properties for Brazilian preschoolers [62].

### Procedures

Recruitment took place at the doors of the schools, and the invitation was made to the children’s guardians when they left the classroom for the class. After written consent, the subsequent steps were scheduled. The first stage was carried out at the child’s home with the completion of questionnaires characterizing the child and your family, economic [47], time outdoors [57] and application of EC-HOME [53] in addition to guidance on the instrument (accelerometer) that the child used to measure the PA level.

The families were instructed about the use of the accelerometer, delivered by a properly trained researcher and positioned on the child’s right hip on every day of use. The family removed the device, placed at 7 am, at 7 pm. The children used the device for 3 days and, if the data were not captured, the use was repeated in the following week.

The second stage was carried out in the school environment, where it was applied by ECERS. To ensure reliability and internal control, only one experienced researcher applied all tests, measures and questionnaires.

### Data analysis

We used the Multi-attribute utility theory (MAUT), a tool used in the setting of the connection and existence of multiple factors in the evaluation process to identify, characterize and combine different variables [63]. Nobre and colleagues [64], in a study using MAUT also presented a similar methodology describing the phases of MAUT:

#### Phase 1: selection of criteria

According to MAUT, selected criteria must faithfully represent what will be assessed and are selected from the literature [65]. Thus, for the physical environmental opportunities for active play, the selected criteria, based on the literature, were: 1-Time the child spends outdoors on weekdays [24, 26, 27, 57, 66], 2-Time the child spends outdoors on weekend days [26, 57]; 3-Presence of internal and external space in the house available to play [23, 33]; 4- External space (patio or court) of the school that allows playing [19, 23]; 5- If the school has a playground (playground) [30, 38, 67].

#### Phase 2: establishing a utility scale for scoring each criterion

Thereafter the criteria selected, we established scores for the selected criteria on the same ordinal scale. Within MAUT it may happen that some selected criteria have different units of measure quantified by means of attributes [65]. In our study, the selected criteria quantified responses using attributes described in the second column of Table 1. In this phase, the responses were converted into numerical variables by means of an ordinal scale. For each answer, a positive value was attributed when the practice was considered favorable and null if the criterion did not characterize physical environmental opportunities for active play.

**Table 1:** Criteria evaluated and possible responses

Criterion	Possible Answers	Pointing
1- Time the child spends outdoors on weekdays (minutes)	35-69 min	0.1
	70-119 min	0.5
	120 min or more	1
2- Time the child spends outdoors on weekend days (minutes)	35-69 min	0.1
	70-119 min	0.5
	120 min or more	1
3- Does the house have an internal environment of at least 30m^2^ per inhabitant and an external space that allows for play?	Yes	1
	No	0
4- Does the school have a space (patio or court) that allows active play?	Yes	1
	No	0
5- Does the school have a park with toys?	Yes	1
	No	0

The first criterion, “Time that the child spends outdoors on days of the week (minutes)” [26, 57, 66], the second criterion, “Time the child spends outdoors on weekend days (minutes)” [26, 57], the third criterion, “House has an internal environment with a minimum of 30m^2^ per inhabitant and an external space that allows play” [24, 33] and the fourth criterion, “School has space (patio or court) that allows active play” [19, 23] .

The fifth criterion, “School has a park with toys” [30, 38, 67], scored 1 the child who studied at a school that had a park with toys that encourage gross motor coordination and 0 the school that did not have a park with toys, according to ECERS criteria [59]. Thus, based on phase 1, the child with the highest score in the multicriteria analysis of physical environmental opportunities for PA is the one who spent 120 min or more playing outdoors on weekdays and on weekends. This child resided in a house with an internal space of at least 30 m^2^ per inhabitant and with a yard or external space that allowed active play and studied in a school that contained a patio or court that allowed movement and a park with toys. Table 1 presents the criteria with the possible scores.

#### Phase 3: determination of the weight for each criteria of physical environmental opportunities

The number represents the importance of each criterion is weight. If the decision maker understands that one criterion is more relevant than the other (supported by the literature or in the opinion of experts on the subject), it will have greater weight [63]. For the research, equal weights were used for the different criteria, assuming that each selected factor has the same degree of relevance in the process of physical environmental stimulation opportunities for PA practice experienced by children.

#### Phase 4: calculation of the multicriteria index of physical environmental opportunities

The multicriteria index of physical environmental opportunities refers to the weighted sum of the evaluations of the different criteria. In our study, the weights considered for each criterion were the same (phase 3); therefore, to calculate the multicriteria index of physical environmental opportunities, an average of the evaluations of all criteria were established for each participating child. It is observed, in eq. 1, how this calculation was made (n = number of criteria evaluated):

1 $${\displaystyle \begin{array}{l} Multicriteria\ indexof\ physical\ environmental\ {opportunities}_{child\ i}=\\ {} Evaluation\ criterion\ {1}_{child\ i}{weight}_{criterion\ 1}+.\dots + Evaluation\ criterion\ {n}_{child\ i}\;{peso}_{criterion\ n}\end{array}}$$

#### Phase 5: validation of results

At this moment, we verified whether the multicriteria methodology carried out meets the objective [51, 53]. Our study checked the relationship and contribution of the index to explain objectively the level of PA of sedentary PA, intensity of light, moderate, vigorous, MVPA and classification as “active” and “insufficiently active” [47]. Thus, a correlation analysis was carried out between the multicriteria index of physical environmental opportunities and the PA intensities collected by the accelerometer.

The Excel Program (version-2010) was used to formulate the multicriteria model, later, for the validation stage; the data were transferred to the Statistical Package for the Social Sciences (version-23.0), to perform Pearson’s correlation analysis and simple regression analysis (*p* < 0.05). After applying Shapiro Wilk test on the multicriteria index of physical environmental opportunities, we found that the variable had a normal distribution, performing a subsequent Pearson correlation analysis. Then, we analyzed those variables that showed a correlation above 0.20 by simple linear regression analysis in order to verify how much the multicriteria index of physical environmental opportunities could explain the PA intensities.

## Results

Table 2 shows the participants characteristics. Participated in this study, 51 preschoolers enrolled in 9 public Municipal Early Childhood Education Centers, with an average age of 4.5 years (SD ± 0.60), with a slight predominance of boys (53%). Most of the children’s families were made up of couples living with partners and more than half of the mothers had 8 years or more of schooling (65.4%). Most families belonged to the lower middle class (class C, 63.4%) and lived in houses classified as medium stimulation environments (78%). Of the participating children, most did not perform systematized PA in spaces such as clubs and similar; many accumulated 180 min/day of PA and just over half of the children accumulated 60 min/day in MVPA. The majority (64.7%) studied in the partial school shift, which totalized an average time of 4 h and 30 min a day in preschool (Table 2). Despite the average time of MVPA of the respondents meeting the WHO recommendations (WHO 2019), the average time in sedentary PA objectively measured ads up to almost 7 h a day.

**Table 2:** Characterization of participants (*n* = 51) and correlation with the multicriteria index of physical environmental opportunities

Characteristics	N(%)	R	***p*** value
Age Mean (min-max)	5 (3-5)	0.066^a^	0.644
Shift		1.208^c^	1.364
Integral	18 (34.60)		
Evening	16 (30.80)		
Morning	18 (34.60)		
Sex		3.029^b^	0.082
Male	28 (53.80)		
Female	24 (46.20)		
Maternal schooling		0.023 ^c^	1.019
Fundamental	14 (21.20)		
High school	20 (38.50)		
University education	7 (13.50)		
Economic Classification^2^		1.787 ^c^	0.344
Class B	14 (26.90)		
Class C	33 (63.40)		
Class D and E	5 (2.80)		
Quality of the school environment Score Mean(min–max)	2.57 (1.90-2.92)	− 0.050	0.730
Home Classification			
High stimulation	10 (19.20)		
Medium Stimulation	42 (78.80)		
Classification Active or Insufficiently active (180 min at any intensity)		1.000^b^	0.635
Active	49 (96.10)		
Insufficiently active	2 (1.10)		
Classification Level PA (WHO 2019)^1^		8.241^b^	0.004*
Ative	28 (54.90)		
Insufficiently active	23 (45.10)		
Sedentary time ^3^ minutes/day	393.991(±45.79)	−0.157 ^a^	0.270
Light intensity^3^ minutes/day	189.34(±34.86)	0.177 ^a^	0.215
Moderate intensity^3^ minutes/day	40.26(±10.34)	0.347 ^a^	0.013*
Vigorous intensity^3^ minutes/day	20.09(±6.47)	0.362 ^a^	0.009*
MVPA intensity^3^ minutes/day	60.37(±14.53)	0.408 ^a^	0.003*
Light to vigorous sum^3^ minutes/day	249.70(±44.98)	0.269^a^	0.056

^1^ Classification Level PA (physical activity) 180 min/day at any intensity, with 60 min/day being (MVPA). ^2^ Class B (higher economic class) to D-E (lower economic class). ^3^ Mean (minutes/day) and Standard Deviation values ^a^ Pearson’s correlation; ^b^ chi-squared test or fisher exact test. ^c^ T test for independent samples. * *p* < 0.05

**Fig. 1:**
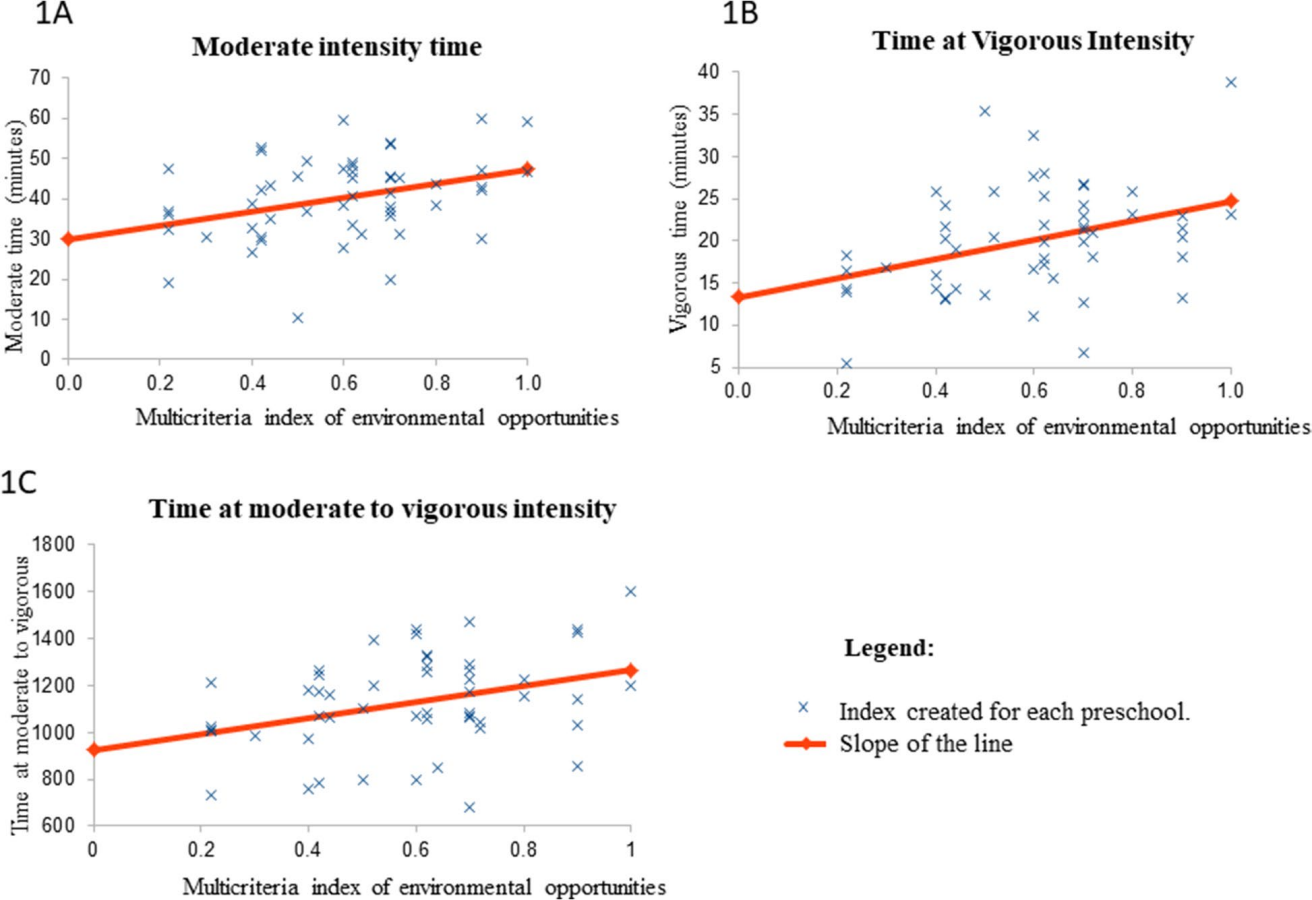
Correlation graphs between the multicriteria index of physical environmental opportunities and physical activity (PA) intensities

The multicriteria index of physical environmental opportunities was calculated following the phases described in the methodology section. Figure 1 shows the validation phase that represents the correlation between the multicriteria index of physical environmental opportunities and the PA intensities. In graph 1A, children who obtained a higher multicriteria index of physical environmental opportunities had a longer at moderate intensity. Therefore, the correlation was statistically significant, positive and moderate. In 1B graph, the children who obtained a higher multicriteria index of physical environmental opportunities, obtained a longer time in the vigorous intensity, statistically significant, positive and moderate correlation. In 1C, the children who obtained the highest multicriteria index had a longer time in the MVPA intensity, statistically positive correlation, significant and moderate.

**Fig. 2:**
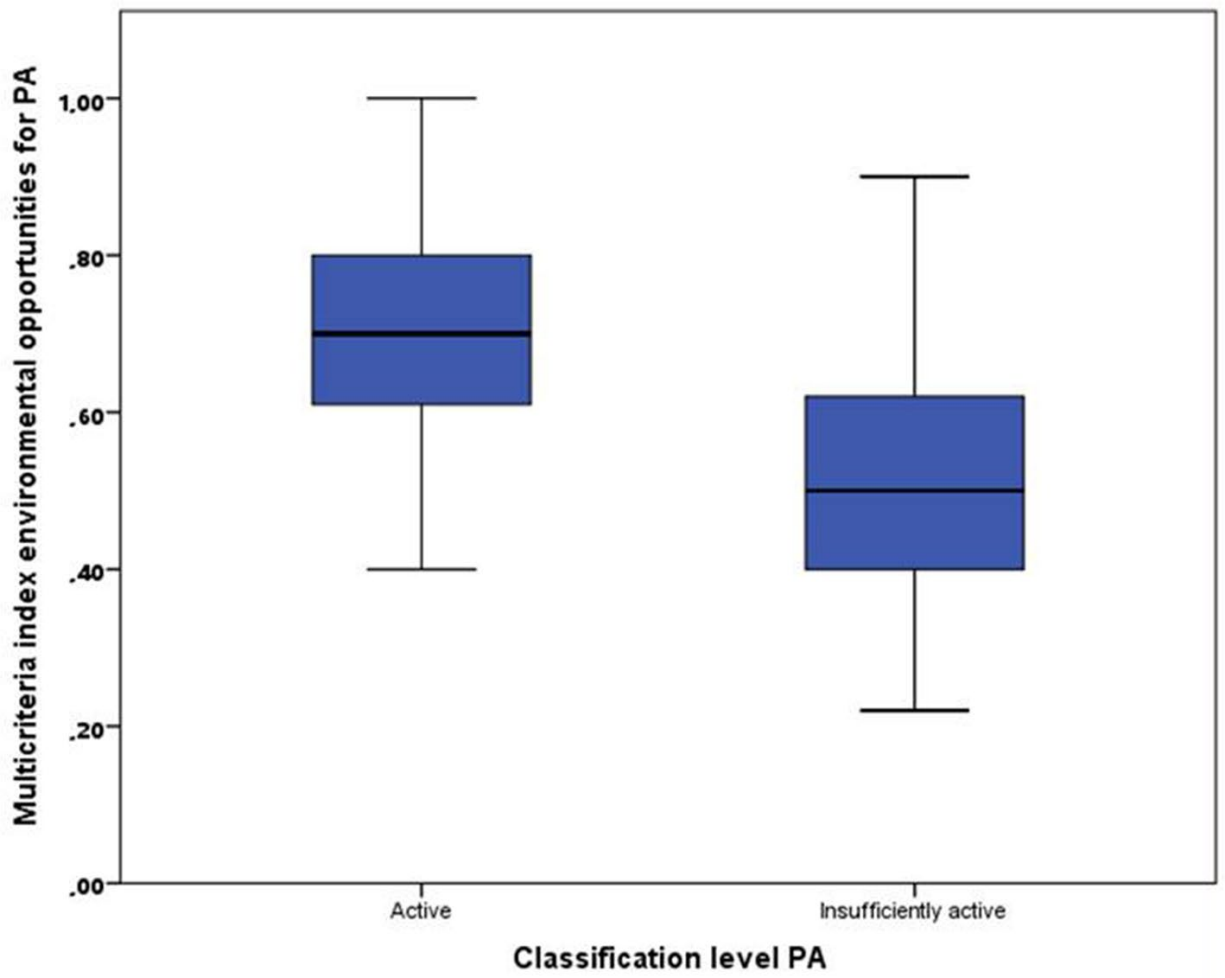
Mean difference between the physical activity classification and the multicriteria index of physical environmental opportunities

In Fig. 2, the boxplot shows the relationship between the multicriteria index of physical environmental opportunities and the PA classification of children as active and insufficiently active. Thus, children who had more quality physical environmental opportunities for PA (higher value in the multicriteria index of physical environmental opportunities) were classified as active. In this sense, the relationship was positive, significant (*p* = 0.001) and moderate (x^2^ = 0.44).

We also showed linear regression with the outcome variable multicriteria index of physical environmental opportunities as a validation of the multicriteria index in Table 3. This study developed an index to measure the physical environmental opportunities of free active play for preschoolers of middle-income countries. Thus, having a higher value in the multicriteria index of physical environmental opportunities explained 12% of the moderate intensity (*p* = 0.013), 13% of the vigorous intensity (*p* = 0.009) and 16% of MVPA (*p* = 0.003). In addition, have physical environmental opportunities to practice higher quality PA explained 20% of the active classification (*p* = 0.001).

**Table 3:** Linear regression of physical activity intensities with the outcome variable of physical activity opportunities included in the multicriteria index of physical environmental opportunities

Physical activity intensities	Outcome variable of physical activity opportunities			
	R^**2**^	b ± SE	Β	***P***
Sedentary time (minutes/day)	0.025	−0.001 ± 0.001	−0.157	0.270
Light intensity (minutes/day)	0.031	0.001 ± 0.001	0.177	0.215
Moderate intensity (minutes/day)	0.120	0.007 ± 0.003	0.347	0.013*
Vigorous intensity (minutes/day)	0.131	0.004 ± .0.362	0.362	0.009*
MVPA intensity (minutes/day)	0.166	0.002 ± 0.408	0.408	0.003*
Light to vigorous sum (minutes/day)	0.072	0.001 ± 0.001	0.269	0.056
Classification Active or insufficiently active (WHO 2019)	0.200	0.184 ± 0.053	0.447	0.001*

*MVPA* Physical activity. R^2^ = coefficient of determination (r square adjusted). b = Non-standard coefficient; ±SE = Standard error. β = Standardized Beta. *P* value = *Statistical significance *p* < 0.05

The effect size was calculated using Cohen’s d [68] which considers Cohen’s d = 0.20, 0.50, and 0.80 to interpret observed effect sizes as small, medium, or large, respectively. Thus, for moderate intensity, the study showed a power of 0.86 (considering an alpha error of 0.05, effect size of 0.13). For vigorous intensity the power was 0.86 (Alpha error of 0.05, effect size of 0.14). For MVPA intensity, the power was 0.86 (effect size of 0.19) and for Classification Level PA, the power presented was 0.86 (effect size of 0.25) [68].

## Discussion

Our data revealed a positive relationship of the multicriteria index of physical environmental opportunities with the MVPA intensity. In addition, the physical environmental opportunities for PA explained 20% of the preschooler’s classification as active and 16% of the time in MVPA. It is noteworthy the relationship was moderate [69] and is in line with previous studies with similar methodology involving child development [64]. This study also used multicriteria index, associated it with domains of child development, and found moderate relationships [64].

The preschool phase is a sensitive moment for the experience of PA. In addition, enables the development of motor competence [ 5, 70], which may facilitate engagement in sports and healthy lifestyle maintenance, and creates life habits that tend to last in later stages of life [1]. A previous study using direct PA measurement in Sweden preschoolers evidenced that the structural characteristics of the preschool, e.g., formalized PA policy and more time spent outdoors, were positively associated with PA of children [71]. Thus, formalized PA policies and outdoor time can be important for promoting children’s PA during preschool hours [20]. Moreover, previous studies have advocated the expansion of the time in outdoor recreation associated to a structured PA in the preschool environment [43, 72]. Thus, the promotion of PA and opportunities to encourage the natural desire for movement beginning them early in life is beneficial [10].

Studies about PA intensities for preschoolers have focused on MVPA [73]. Of note, the basis for prioritizing MVPA is probably the beneficial impact pointed on improvement of health-related physical fitness conditions [2, 74], cognitive development [75]and increased motor competence [76]. In addition, the preschool space having parks containing toys and equipment as well as a patio enabling the preschoolers to increase the physical environmental opportunities for active play during recreation time [20, 67, 71]. With this regard, despite the educational legislation does not make the presence of physical education professionals mandatory in the context of preschool [77, 78], Brazilian preschoolers who have the presence of a physical education professional probably have better motor skills [77]. Given the above, we hypothesize that the presence of the park at the preschool could be determinant to increase the PA opportunities, especially in the MVPA intensity [67, 71].

Our multicriteria index of physical environmental opportunities also pointed the importance of the home environments [7]. The family environment plays an important role to provide opportunities for physical activities [66, 79]. In particular, playing outdoors requires social support and parental supervision [80]. In addition, because parental restrictions can prevent participation in PA and outdoor play in preschoolers [81], our data reinforce the importance of external and internal space for active play at home, since most of the responsibility for promoting healthy behaviors and PA practices currently falls on families [82].

About the evaluation of the quality of home, our data showed that more than half of preschoolers live in medium stimulus environments [53] and belong to class C, e.g., extract that comprises the lower middle class. Thus, for families whose houses do not have external and internal spaces that allow active play [7], the presence of parks and outdoor leisure areas in the neighborhood daycare environment seems to be crucial for children to increase the level of PA especially children whose home environment may not be conducive to activity [30].

Considering PA time including all the intensities, 96.1% of the Brazilian preschoolers accumulated 180 min/day of PA. Furthermore, the quality of environmental opportunities for active play seemed to contribute substantially to the acquisition of moderate, vigorous and MVPA intensities, and for the preschooler to become physically active. Surprisingly our data showed that the majority of the Brazilian preschoolers reached the recommended minimum daily PA. In this sense, the daily PA time in Brazilian preschoolers was higher compared to Chinese preschoolers (83.8%) [83], with their accumulated daily PA time more likely due to time spent at light intensity [23].

We suppose that the environmental factors together assessed by the multicriteria index of physical environmental opportunities corroborate the reach of MVPA intensity. Namely, the mean MVPA intensity values of active preschoolers (Mean ± SD = 70.68 min ± 9.09) and of insufficiently active preschoolers (47.81 min ± 8.85). Active preschoolers scored higher on the multicriteria index of physical environmental opportunities (0.67 point ±0.18) compared to those who were insufficiently active (0.49 point ±0.18). Other studies investigated preschoolers with the same time points for the PA classification [52] and our data showed that the percentage of Brazilian preschoolers (54.90%) who meet the daily guidelines [52] was higher than Canada (13.7%) [84] and Sweden (33%) preschoolers [17].

Collectively, our results are in line with Tucker and colleagues study [72] and support the implementation of opportunities that increase children’s access to outdoor play, as well as ample spaces, both in preschool ambient [20, 43] and in other places such as the home environment [26, 31, 33], in order to provide PA opportunities using body movement experiences [43]. In this sense, the multicriteria analysis meets a current demand in the care of the pediatric population, regarding the construction of parameters that indicate physical environmental opportunities for active play and physical activity level in preschoolers.

Our study has strengths and limitations. The cross-sectional design of the present study does not allow inferring cause and effect. Aspects related to social modeling, parental encouragement, and logistical support for PA should be considered in future works. In addition, our sample seemed to be a very active sample, which limits generalizability of the findings.

Although accelerometry is a direct measure of PA intensity among preschoolers [49] and used in many studies [82], accelerometry is a measure unable to detect accurately PA intensity in activities with significant upper body movements. However, the direct measurement of daily PA level avoided the risk of bias related to the self-reported measures such as memory difficulties and social desirability.

An important limitation of this study is that the ECERS questionnaire was not validated for Brazilian preschoolers. However, this questionnaire [59] is a well-known international instrument translated to Portuguese [60] and used by different Brazilian studies with preescholers [60, 61] with established psychometric properties [62]. Of note, although previous international groups from different countries, including Brazil, used the Home Observation for Measurement of the Environment - HOME Inventory, the instrument has not been subject to an analysis of measurement equivalence/invariance cross-culturally [55]. However, we noteworthy that the HOME Inventory has been used worldwide to evaluate the home environment in both international [54] and transcultural studies [55], presenting psychometric characteristics that were investigated in Brazilian preschoolers sample [56].

Moreover, we used questionnaires [57] that allowed the assessment of the quality of the home [53] and school [59] environments allowing the elaboration of the multicriteria index of physical environmental opportunities to evaluate PA opportunities.

## Conclusions

Physical environmental opportunities were determinant for the higher intensities of PA. Therefore, playing outdoors, living at home with a yard and indoor space, studying in schools with a patio and playground seem to favor the possibilities for preschoolers to experience MVPA.

## Data Availability

The datasets used and/or analyzed during the current study are available from the corresponding author on reasonable request.

## Abbreviations

ABEP: Associação brasileira de estatística e pesquisa
EC-HOME: Early Childhood Home Observation for Measurement of the Environment
ECERS: Early Childhood Environment Rating Scales
MAUT: Multi-attribute utility theory
MVPA: Moderate to vigorous physical activity
PA: physical activity
UFVJM: Universidade Federal dos Vales do Jequitinhonha e Mucuri
WHO: World Health Organization
